# CYP2C19 Genetic Variability, ADP-Induced Whole-Blood Platelet Aggregation, and Clopidogrel Response in an Exploratory Retrospective Cohort of Acute Ischemic Stroke

**DOI:** 10.1177/10760296261486174

**Published:** 2026-09-01

**Authors:** Soumya Ravichandran, Cattien Phan, Dawn M. Meyer, Amirmohammad Eghbalnejadmofrad, Amirhossein Akhavan-Sigari, Seyed Behnam Jazayeri, Sanad Batarseh, Marissa D’Souza, Tiffany Hu, Lovella Hailey, Kunal Agrawal, Royya F. Modir, Thomas Hemmen, Brett C. Meyer, Reza Bavarsad Shahripour

**Affiliations:** 1 Department of Neurosciences, Comprehensive Stroke Center, 8784 University of California San Diego, San Diego, CA, USA; 2 Department of Neurology, 12349 University of Virginia School of Medicine, Charlottesville, VA, USA; 3 Department of Neurological Surgery, 23386 Mayo Clinic, Phoenix, AZ, USA

**Keywords:** acute ischemic stroke, clopidogrel resistance, pharmacogenomics, personalized antiplatelet therapy, platelet aggregation testing

## Abstract

**Background:**

Patient response to clopidogrel varies due to CYP2C19 genetic polymorphisms. Impaired metabolism has been linked to an increased risk of recurrent ischemic events. The purpose of this study was to examine CYP2C19 phenotypes and the impact on ADP aggregation in whole blood platelet aggregometry (WBPA) in patients with acute ischemic stroke (AIS).

**Methods:**

Patients with AIS or transient ischemic attack (TIA) from two academic comprehensive stroke centers between January 2022 and May 2025 were retrospectively reviewed. Inclusion criteria required both CYP2C19 genotyping and WBPA. Categorical variables were compared using chi-square test and continuous variables using Mann-Whitney U test. Multivariable logistic regression identified independent predictors of inadequate platelet inhibition defined by ADP aggregation threshold.

**Results:**

Among 112 patients (metabolizers: N=62, non-metabolizers: N=50), there was no correlation between CYP2C19 metabolizer phenotype and ADP-induced platelet aggregation (p=0.49). The study found substantial racial (p=0.012) and Hispanic ethnic (p=0.001) differences in metabolizer phenotype. Inadequate platelet inhibition was also associated with age (p=0.020) loading phase (p=0.04), underscoring the need to consider demographic variables, patient characteristics, and the timing of platelet function testing when interpreting platelet reactivity results and making clinical decisions.

**Conclusion:**

This study found no significant association between an individual’s CYP2C19 metabolizer status and ADP-induced platelet aggregation. The results of this study may help advance understanding of pharmacogenomics, but care must be taken when interpreting them due to this study’s limitations. These findings should be confirmed in a future study.

## Introduction

Clopidogrel is an oral prodrug widely used in the secondary prevention of non-cardioembolic ischemic stroke, transient ischemic attack (TIA), and other atherothrombotic conditions.^1,2^ Following absorption, the drug undergoes CYP2C19-dependent hepatic biotransformation to generate an active thiol metabolite, which irreversibly binds the platelet P2RY12 receptor and inhibits adenosine diphosphate (ADP)-mediated platelet aggregation.^1^ Whole blood platelet aggregometry (WBPA) provides a direct measure of this pharmacodynamic effect and is used clinically to assess the degree of platelet inhibition.^3^ Adequate platelet inhibition is essential to clopidogrel’s therapeutic effect, and failure to achieve sufficient suppression of platelet reactivity has been associated with an increased risk of recurrent ischemic events.

Despite its widespread use, clopidogrel exhibits substantial interindividual variability in metabolism and platelet inhibitory response, driven primarily by CYP2C19 genetic polymorphisms.^1^ Loss-of-function (LOF) alleles, characteristic of non-metabolizer phenotypes, reduce enzymatic activity and diminish active metabolite production, while the gain-of-function (GOF) alleles, associated with metabolizer phenotypes, enhance metabolic capacity. Non-genetic factors including proton pump inhibitor use, diabetes mellitus, chronic kidney disease, and advanced age further modulate drug response.^2^ Notably, CYP2C19 LOF allele frequencies vary substantially across racial and ethnic populations, with certain variants occurring at higher prevalence in Hispanic, Asian, and African American populations, representing a dimension of variability that remains underappreciated and incompletely characterized in the existing literature.^4^

The clinical significance of non-metabolizer phenotypes has been most robustly established in cardiac populations undergoing percutaneous coronary intervention (PCI), where impaired clopidogrel metabolism is independently associated with adverse cardiovascular outcomes.^3,5^ Evidence in ischemic stroke populations remains far less conclusive, with multiple studies reporting weak or inconsistent associations between CYP2C19 genotype and recurrent cerebrovascular events.^2,6,7^ Platelet function testing, including the VerifyNow P2Y12 assay and WBPA, has been used to assess platelet inhibitory response to clopidogrel.^8,9^ However, no universally validated cutoff value has been established across these platforms.^8,10^ Furthermore, concordance between CYP2C19 genotype and platelet reactivity phenotype as measured by these assays remains inconsistent, underscoring the limitations of relying on genotype alone to predict platelet inhibition.^2,11^ Hispanic and other minority populations remain markedly underrepresented in existing pharmacogenomics studies, limiting the generalizability of current evidence to diverse real-world stroke cohorts.^4^

There remains a critical need to evaluate how CYP2C19 metabolizer and non-metabolizer phenotypes interact with demographic factors and platelet function results in real-world stroke cohorts. The present study addresses this gap using a retrospective cohort of acute ischemic stroke (AIS) and TIA patients from two comprehensive stroke centers serving a demographically diverse population with substantial Hispanic representation. Two specific aims were pursued: first, to determine whether CYP2C19 metabolizer phenotype correlates with age, gender, and race/ethnicity; and second, to examine the relationship between CYP2C19 metabolizer phenotype and ADP-induced platelet reactivity. These findings provide real-world evidence bearing on the reliability of CYP2C19 metabolizer phenotype as a surrogate for platelet reactivity phenotype in a diverse stroke population, with direct implications for individualized antiplatelet decision-making.

## Methods

### Study Design and Data Source

This was a retrospective cohort study of adult patients presenting with AIS or TIA at two comprehensive stroke centers within the UC San Diego Health System between January 1, 2022 and May 31, 2025. Clinical data was extracted from the EPIC electronic health record system. The study was approved by the UCSD Institutional Review Board (IRB #813094) and an informed consent waiver was granted given the retrospective study design and use of de-identified data.

### Patient Selection

Patients were eligible for inclusion if they had a confirmed diagnosis of AIS or TIA, underwent CYP2C19 genotyping, and had ADP-mediated platelet function testing performed via WBPA while on therapeutic clopidogrel. Therapeutic clopidogrel exposure was defined as either a 300 mg loading dose with platelet function testing performed after 8 hours, or a minimum of 7 consecutive days of maintenance therapy at 75 mg daily. Patients were excluded if platelet function testing was performed outside the defined pharmacodynamic window, if clopidogrel exposure was inadequate or uncertain, if testing was performed prior to clopidogrel initiation, if IV P2Y12 inhibitor use was documented, or if critical clinical, laboratory, or genotyping data were missing.

### Demographic Clinical Data

Clinical data extracted from the electronic health record included demographic variables (age, sex, race, and self-reported ethnicity) and comorbid conditions (hypertension, diabetes mellitus, hyperlipidemia, coronary artery disease, and prior ischemic stroke or TIA). Antiplatelet regimen at the time of platelet function testing, including use of dual antiplatelet therapy (DAPT), was determined from pharmacy records.

### Laboratory Assessments

CYP2C19 genotyping was ordered as part of routine clinical practice and performed at the UCSD Molecular Diagnostics Laboratory using a clinically validated assay. The institutional panel interrogated the *2 and *3 LOF alleles and the *17 GOF allele. Alleles associated with reduced or absent enzymatic activity not captured by this panel, including *4, *5, *6, *8, *10, and *11, were not assessed. CYP2C19 metabolizer phenotypes were assigned according to Clinical Pharmacogenetics Implementation Consortium (CPIC) definitions and classified into two groups for analysis.^12^ Patients carrying at least one LOF allele, classified as poor or intermediate metabolizers, were designated as non-metabolizers. Patients without LOF alleles, classified as normal, rapid, or ultrarapid metabolizers, were designated as metabolizers.

Whole blood platelet aggregometry was used to assess platelet function by measuring ADP-induced platelet aggregation. In patients with multiple WBPA, we used the value obtained at the time of clopidogrel administration for analysis, with a maximum time window of 24 hours. Platelet reactivity phenotype was classified using a validated institutional cutoff established by the UCSD Platelet Function Laboratory: ADP-induced aggregation < 40 units was classified as adequate platelet inhibition, ADP-induced aggregation ≥ 40 units were classified as having inadequate platelet inhibition. This threshold is in line with existing clinical cut points for impedance-based platelet function testing.^13^ The Roche Multiplate Analyzer was not used in a dedicated research trial but was used routinely in clinical practice for stroke patients on antiplatelet therapy. For all tests, the certified laboratory scientists analyzed the samples at a standard ADP concentration (6.4 μM), yielding consistent, reliable results for adequate clinical interpretation.

### Statistical Analysis

Baseline characteristics were summarized using descriptive statistics. Continuous variables were reported as mean ± standard deviation or median with interquartile range and compared between groups using the Mann-Whitney U test. Categorical variables were reported as frequencies and percentages and compared using the chi-square test or Fisher’s exact test as appropriate.

We identified the independent predictors of inadequate platelet inhibition using multivariable logistic regression. Besides the factors that were significant in the bivariate analyses (p < 0.20), the following variables were selected a priori due to their clinical relevance, biological importance, and impact on clopidogrel metabolism: race, hyperlipidemia, diabetes, metabolizer phenotype and dosing phase. Hyperlipidemia was included because statins are associated with hyperlipidemia and may influence clopidogrel metabolism through the CYP450 pathway. Diabetes mellitus was not part of the final model as it was not significantly linked to the outcome in the preliminary bivariate analysis (p=0.251), and to avoid overfitting. To assess the linearity assumption for age, the Box-Tidwell transformation was applied, which showed an insignificant interaction term (p=0.946), making it suitable for logistic regression. The race variable was simplified into White and non-White categories to enhance estimated stability. Due to the exploratory nature of this analysis, we did not perform multiple comparisons. All statistical tests were two-tailed, with p-values < 0.05 indicating statistical significance.

## Results

### Patient Population

**Figure 1.**
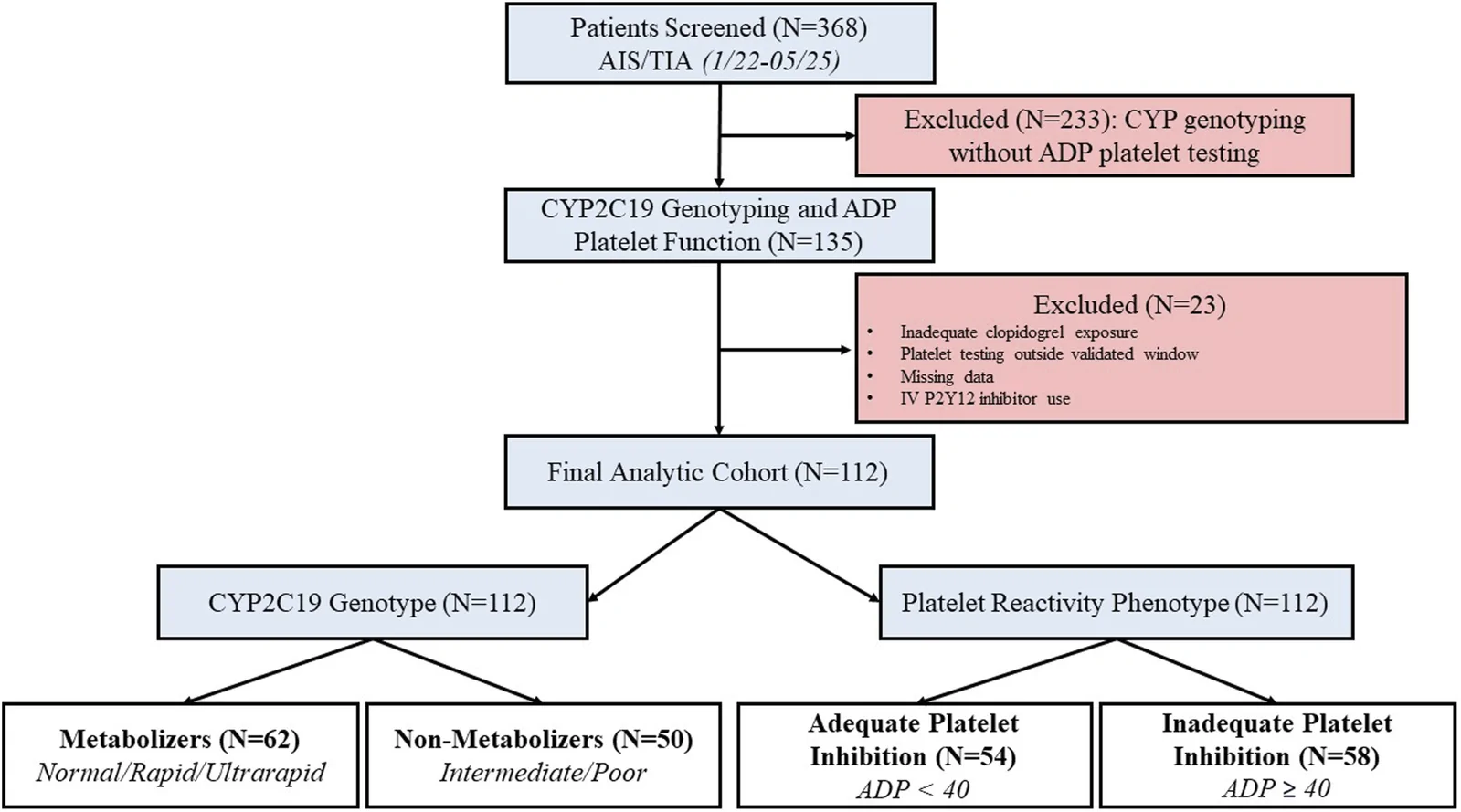
CONSORT Flow Diagram of Patient Enrollment and Study Progression

Patient enrollment and study progression are summarized in Figure 1. Of the 368 patients initially screened with acute ischemic stroke or TIA, 112 patients met all inclusion criteria and comprised the final analytic cohort. The exclusion of the remaining 256 patients were due to the absence of either CYP2C19 genotype testing or WBPA within the predefined pharmacodynamic window. By CYP2C19 metabolizer phenotype, 62 patients (55.4%) were classified as metabolizers and 50 patients (44.6%) as non-metabolizers. By platelet reactivity phenotype, 54 patients (48.2%) displayed adequate platelet inhibition and 58 patients (51.8%) demonstrated inadequate platelet inhibition. Most patients were on DAPT at the time of assessment (metabolizers: 72.6%, non-metabolizers: 70.0%, total: 71.4%).

### Baseline Characteristics

Baseline demographic and clinical characteristics were generally well-balanced between the metabolizer and non-metabolizer phenotype and are summarized in Table 1. The mean age of the cohort was 68.9 ± 11.0 years (metabolizers: 67.9 ± 11.1; non-metabolizers: 69.9 ± 13.6; p=0.39). Most baseline clinical characteristics were well-balanced between groups, including sex, platelet reactivity phenotype, DAPT use, hypertension, diabetes mellitus, coronary artery disease, and prior stroke or TIA (all p > 0.05). Dyslipidemia was numerically higher among metabolizers compared to non-metabolizers (93.5% vs. 84.0%; p=0.06), though this did not reach statistical significance. Race differed significantly between metabolizer and non-metabolizer groups (p=0.012) and is explored in detail in subsequent analyses. Hispanic ethnicity was also significantly more prevalent among metabolizers compared to non-metabolizers (41.9% vs. 12.0%; p=0.001).

**Table 1.** **Demographic and Baseline Clinical Characteristics**. The baseline characteristics are provided for the total cohort as well as the metabolizer and the non-metabolizer groups.

Variable	Metabolizer (N=62)	Non-metabolizer (N=50)	Total (N=112)	p-value
**Age, mean (SD)**	67.9 (11.1)	69.9 (13.6)	68.9 (11.0)	0.39
**Sex**	​	​	​	0.55
Female	28 (45.2%)	19 (38.0%)	47 (42.0%)	​
Male	34 (54.8%)	31 (62.0%)	65 (58.0%)	​
**Race**	​	​	​	0.012
African American	6 (9.7%)	7 (14.0%)	13 (11.6%)	​
Asian	2 (3.2%)	9 (18.0%)	11 (9.8%)	​
White	29 (46.8%)	24 (48.0%)	53 (47.3%)	​
Other	25 (40.3%)	8 (16.0%)	33 (29.5%)	​
**Ethnicity**	​	​	​	0.001
Hispanic	26 (41.9%)	6 (12.0%)	32 (28.6%)	​
Non-Hispanic	35 (56.5%)	41 (82.0%)	76 (67.9%)	​
Unknown	1 (1.6%)	3 (6.0%)	4 (3.5%)	​
**Platelet Reactivity Phenotype**	​	​	​	0.24
Adequate Platelet Inhibition	33 (53.2%)	21 (42.0%)	54 (48.2%)	​
Inadequate Platelet Inhibition	29 (46.8%)	29 (58.0%)	58 (51.8%)	​
**DAPT**	45 (72.6%)	35 (70.0%)	80 (71.4%)	0.77
**Hypertension**	52 (83.9%)	43 (86.0%)	95 (84.8%)	0.76
**Diabetes Mellitus**	32 (51.6%)	25 (50.0%)	57 (50.9%)	0.86
**Dyslipidemia**	58 (93.5%)	42 (84.0%)	100 (89.3%)	0.06
**Coronary Artery Disease**	22 (35.5%)	21 (42.0%)	43 (38.4%)	0.62
**Prior Stroke/TIA**	31 (50.0%)	26 (52.0%)	57 (50.9%)	0.73

Abbreviations: DAPT, dual antiplatelet therapy; TIA, transient ischemic attack; SD, standard deviation.

### Association Between CYP Metabolizer Phenotype and ADP Aggregation

**Figure 2.**
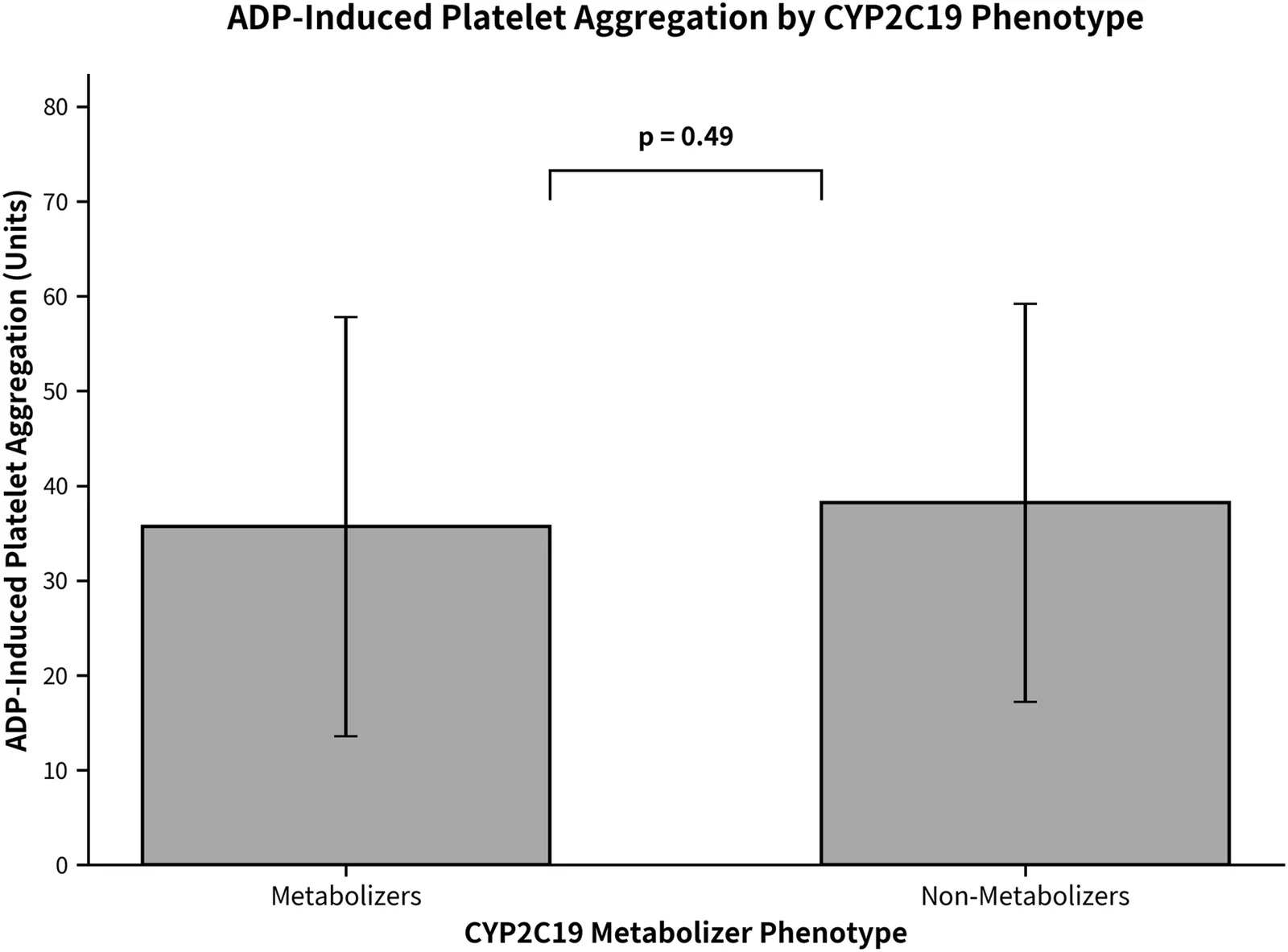
**Association Between CYP2C19 Metabolizer Phenotype and ADP-Induced Platelet Aggregation**. The mean ADP-induced platelet aggregation was slightly elevated in non-metabolizers, but there was no significant difference (p = 0.49). This variability among individuals suggests that, in this context, CYP2C19 metabolizer status might not be a reliable predictor, as other factors may be involved in platelet inhibition.

The difference in platelet aggregation induced by ADP between the metabolizer and non-metabolizer phenotypes was not significantly different (35.7 ± 22.1 vs. 38.2 ± 21.0 units, respectively, p=0.49). This implies that, in this population, platelet inhibitory response cannot be simply predicted by CYP2C19 metabolizer phenotype alone (Figure 2). In particular, there was significant disagreement between the CYP2C19 metabolizer phenotype and platelet reactivity phenotype: 29 of 62 metabolizers (46.8%) had inadequate platelet inhibition, whereas 21 of 50 non-metabolizers (42.0%) had adequate platelet inhibition. There was no difference in the mean ADP-induced platelet response between metabolizers (35.7 ± 22.1 units) and non-metabolizers (38.2 ± 21.0 units) (p=0.49). The present data suggest that the CYP2C19 metabolizer phenotype is not a good predictor of platelet reactivity in this patient population (Figure 2).

### Race, Ethnicity, and CYP2C19 Metabolizer Phenotype

CYP2C19 metabolizer phenotype distribution differed significantly across racial groups (p=0.009), with Asian patients demonstrating the highest prevalence of non-metabolizer phenotype (9 of 11, 81.8%), compared to African American (7 of 13, 53.8%), White (24 of 53, 45.3%), and other racial groups (8 of 33, 24.2%) (Table 2). Across ethnicities, CYP2C19 metabolizer phenotype also showed a significant difference (p=0.001) with the Hispanic group showing the highest prevalence for the metabolizer phenotype (26 of 32, 81.3%) (Table 1). Despite these genetic differences, platelet reactivity phenotype did not differ significantly across racial groups (p=0.76), which showed similar prevalences for the adequate and inadequate platelet inhibition groups (Table 2). These findings further support the dissociation between CYP2C19 metabolizer phenotype and platelet reactivity phenotype observed in this cohort.

**Table 2.** Race-Stratified Distribution of CYP2C19 Metabolizer Phenotype and Platelet Reactivity Phenotype

Variable	African american (N=13)	Asian (N=11)	White (N=53)	Other (N=33)	p-value
**CYP2C19 Genotype Status**	​	​	​	​	0.009
Metabolizers	6 (46.2%)	2 (18.2%)	29 (54.7%)	25 (75.8%)	​
Non-Metabolizers	7 (53.8%)	9 (81.8%)	24 (45.3%)	8 (24.2%)	​
**Platelet Reactivity Phenotype**	​	​	​	​	0.76
Adequate Platelet Inhibition	6 (46.2%)	6 (54.5%)	27 (50.9%)	14 (42.4%)	​
Inadequate Platelet Inhibition	7 (53.8%)	5 (45.5%)	26 (49.1%)	19 (57.6%)	​

### Predictors of Inadequate Platelet Inhibition

The mean baseline ADP aggregation was 40.16±19.86 units in patients who received a loading dose (N=56,) and 33.54±22.83 units in patients on steady maintenance therapy (N=56) (p=0.10). These mean values were on opposite sides of the clinical threshold of 40 units, therefore, in our multivariable regression model, we adjusted for this variability, rather than assuming equivalence.

Independent predictors of inadequate platelet inhibition status were identified using multivariable logistic regression, with age, sex, race, hyperlipidemia, CYP2C19 metabolizer phenotype, and dosing phase as covariates (Table 3). The analysis showed that age was statistically significantly independently associated with inadequate platelet inhibition: odds of inadequate platelet inhibition increased by 5% per year of age (OR 1.05, 95% CI 1.01–1.09; p=0.016). The dosing phase was also a significant independent predictor; patients evaluated after a loading dose had 2.5 times higher odds of inadequate platelet inhibition than those on maintenance therapy (OR 2.52, 95% CI 1.10–5.80; p=0.04). In addition, there was a trend towards increased odds of inadequate platelet inhibition among patients with hyperlipidemia (OR 4.49, 95% CI 1.00–20.1; p=0.050).

**Table 3.** **Multivariable Logistic Regression Identifying Independent Predictors of Inadequate Platelet Inhibition**. Reference Categories: Male Sex (vs. Female), White Race (vs. Non-White), Hyperlipidemia (vs. Absence of Hyperlipidemia), Non-metabolizer Phenotype (vs. Metabolizer Phenotype), Loading Dose (vs. Maintenance Dose)

Variable	Adjusted OR	95% CI	P-VALUE
Age (Per Year)	1.05	1.01–1.09	0.013
Male Sex	1.68	0.72–3.91	0.228
White Race	0.55	0.23–1.32	0.183
Hyperlipidemia	4.49	1.00–20-13	0.050
Cyp2c19 Metabolizer Phenotype	0.64	0.28–1.45	0.286
Dose Phase	2.52	1.10–5.80	0.040

OR, odds ratio; CI, confidence interval. Bold indicates statistical significance at p < 0.05.

## Discussion

This was a retrospective cohort study of 112 acute ischemic stroke patients and TIA who received clopidogrel at two comprehensive stroke centers. It was designed to assess the association between the CYP2C19 metabolizer phenotype and ADP-induced platelet reactivity by WBPA. The results showed that there was no significant difference in ADP-induced platelet aggregation between the CYP2C19 metabolizer phenotype (35.7 ± 22.1 vs. 38.2 ± 21.0 units; p=0.49). Interestingly, there was a significant discord between genotype and platelet reactivity phenotype in both directions. Further, the prevalence of metabolizer phenotypes was significantly different across races, with the highest prevalence of the non-metabolizer phenotype among patients of Asian origin. The results add to the emerging clinical evidence for the value of CYP2C19 genotype testing in stroke patients, highlighting the importance of non-genetic factors in the variation of clopidogrel response.^14,15^

### CYP2C19 Genotype-Phenotype Discordance

The bidirectional discordance observed between CYP2C19 metabolizer phenotype and platelet reactivity phenotype in our cohort, with nearly half of metabolizers demonstrating inadequate platelet inhibition and over 40% of non-metabolizers having adequate platelet inhibition, highlights the limitations of relying on genotype alone to guide antiplatelet therapy. While prior meta-analyses have demonstrated that CYP2C19 LOF allele carriers face significantly higher rates of recurrent stroke on clopidogrel, these associations reflect clinical outcomes rather than direct pharmacodynamic measurements, and the relationship between genotype and platelet inhibitory response has been shown to be imperfect and context-dependent.^16,17^ In non-East Asian stroke cohorts specifically, evidence linking CYP2C19 metabolizer status to platelet reactivity has been inconclusive, consistent with our pharmacodynamic data.^15^ This dissociation is biologically plausible given that clopidogrel bioactivation involves multiple cytochrome P450 enzymes beyond CYP2C19, and LOF allele carrier status does not uniformly predict the degree of active metabolite generation.^18^ Taken together, these observations suggest that CYP2C19 metabolizer phenotype, while an important pharmacogenomic marker, represents only one determinant of the functional platelet response to clopidogrel in a clinically heterogeneous stroke population.

### Non-Genetic Determinants of Clopidogrel Response

Age was an independent predictor of inadequate platelet inhibition in our cohort, consistent with prior data demonstrating that older patients exhibit higher baseline platelet reactivity and faster platelet turnover independent of metabolizer status.^19,20^ In addition, systemic inflammation, active infection, and uncontrolled diabetes have each been shown to drive platelet reactivity through CYP2C19-independent mechanisms, including upregulation of thromboxane A2 and increased ADP release from activated endothelium.^21^ In an acute stroke population where inflammatory responses are common and comorbidity burden is high, these factors may independently determine platelet inhibition status regardless of metabolizer phenotype.^22^

The timing of platelet function testing also emerged as an independent predictor of inadequate platelet inhibition. Patients tested during the loading-dose phase had more than twice the odds of inadequate platelet inhibition compared with those receiving maintenance therapy. This finding is consistent with previous pharmacodynamic studies demonstrating that ADP-induced platelet aggregation remains significantly higher shortly after a 300 mg clopidogrel loading dose than during steady-state maintenance therapy.^23,24^ Consequently, platelet function testing performed during the acute loading period may overestimate residual platelet reactivity and misclassify patients as having inadequate platelet inhibition. These findings highlight the importance of considering the dosing phase when interpreting WBPA results and suggest that platelet reactivity measured during the loading phase should be interpreted with caution. The trend toward association between hyperlipidemia and inadequate platelet inhibition in our model (OR 4.49; p=0.05) is consistent with this pattern. Together, these findings suggest that clopidogrel variability in platelet inhibition is not a purely pharmacogenomic phenomenon but a clinically complex one, shaped by age, inflammation, and metabolic burden.

### Race, Ethnicity, and CYP2C19 Metabolizer Phenotype Distribution

Despite significant differences in CYP2C19 metabolizer phenotype distribution across racial groups (p=0.009), platelet reactivity phenotype did not differ significantly (p=0.76). This suggests that carrying a LOF allele, which confers a non-metabolizer status, does not uniformly translate into inadequate platelet inhibition across ancestries. The mechanistic basis for this population-specific dissociation is not fully understood, though differences in baseline platelet biology, P2Y12 receptor density, and compensatory enzymatic activity may modulate the functional consequences of LOF alleles across ancestries.^13^ The racial differences in CYP2C19 metabolizer status with Asian patients having the highest prevalence of the non-metabolizer phenotype in this cohort are consistent with known population-level differences in LOF allele frequencies, which are substantially higher in East Asian populations compared to European, African American, and Hispanic groups.^1,25^ The strongest clinical evidence linking CYP2C19 genotype to stroke outcomes (CHANCE-2) enrolled 98% Han Chinese patients, and evidence supporting genotype-guided therapy in other populations remains less certain 26. These findings underscore a critical gap in the pharmacogenomics literature, where Hispanic and other underrepresented populations remain markedly understudied, limiting the generalizability of genotype-guided strategies in diverse cohorts.^4^

### Clinical Implications

Our findings suggest that CYP2C19 metabolizer phenotype alone is insufficient to predict platelet inhibitory response in a real-world stroke cohort. Current CPIC guidelines recommend avoiding clopidogrel in intermediate and poor metabolizers following ACS or PCI, yet explicitly acknowledge that evidence in stroke populations remains limited and that therapy selection should incorporate individual patient factors.^1,27^ Our data reinforce this caution and suggest that functional platelet testing with WBPA identifies patients with inadequate platelet inhibition that genotyping alone would miss. Rather than replacing genotyping, WBPA may be best understood as a complementary tool, with CYP2C19 genotyping serving as an initial screen to identify patients at pharmacogenomic risk, and WBPA confirming functional response in those where clinical uncertainty remains or antiplatelet optimization is being considered. Given that age and dosing phase were identified in this study as significant predictors of inadequate platelet inhibition, the clinical profile of the patient should be considered alongside pharmacogenomic data when optimizing antiplatelet therapy after stroke.

### Limitations and Future Directions

As a retrospective study, this analysis was limited to data available in the electronic health record, and undocumented comorbidities or medications may have introduced misclassification bias. Although the cohort is demographically diverse, the study was conducted within a single health system, which may limit broader generalizability. The institutional CYP2C19 genotyping panel was restricted to the *2, *3, and *17 alleles, and several additional LOF variants (*4, *5, *6, *8, *10, and *11) were not assessed, which may have resulted in misclassification of some patients as metabolizers. Other factors, like smoking, body mass index, renal function, the etiology and severity of the stroke, routine inflammatory markers, and the use of outpatient medications, such as PPIs and statins, were not fully verified by the study. The findings of this study were influenced by these unmeasured variables.

The sample size of 112 patients, while sufficient for exploratory analyses, limits statistical power, particularly in racial and ethnic subgroup analyses. Additionally, there is a small potential risk for selection bias stemming from the exclusion of 256 of the 368 screened patients. However, the reason for exclusion was driven strictly by the absence of either CYP2C19 genotyping or WBPA, reflecting standard clinical ordering practices rather than inherent patient characteristics. While formal comparative baseline analysis was not feasible due to the retrospective nature of data collection on excluded patients, the included cohort is representative of the racially and ethnically diverse population cared for at our institutions. Finally, the absence of longitudinal follow-up data means the clinical consequences of genotype-phenotype discordance on recurrent stroke or TIA risk in this cohort remain unknown.

These findings underscore the need for larger prospective studies with longitudinal follow-up to determine whether genotype-phenotype discordance translates into meaningful differences in clinical outcomes, including recurrent ischemic stroke and TIA. Future work should examine whether antiplatelet medication changes guided by WBPA results are associated with improved outcomes compared to genotype-guided strategies alone. Serial platelet function testing may further clarify whether ADP aggregation values remain stable over time or shift with changes in clinical status or concurrent medications. Larger studies with greater representation of Hispanic and Asian populations are particularly warranted to address the pharmacogenomic gaps identified in this cohort.

## Conclusions

This study demonstrates that CYP2C19 metabolizer status did not appear to significantly affect ADP-induced platelet aggregation. Given the study’s retrospective design and small sample size, the findings should be interpreted with caution. There is a need for additional research, as variations were observed. Further research with larger populations will be crucial for expanding knowledge and developing clinical applications.

## Data Availability

The data that support the findings of this study are available from the corresponding author upon reasonable request and the approval of the Ethics Board. The preliminary results of this study were presented as a poster at the International Stroke Conference 2026 on February 4, 2026 in New Orleans, LA.*
